# Discovery of Cellular Proteins Required for the Early Steps of HCV Infection Using Integrative Genomics

**DOI:** 10.1371/journal.pone.0060333

**Published:** 2013-04-12

**Authors:** Ji Hoon Park, Solip Park, Jae-Seong Yang, Oh Sung Kwon, Sanguk Kim, Sung Key Jang

**Affiliations:** 1 Division of Molecular and Life Science, Pohang University of Science and Technology, Pohang, Korea; 2 School of Interdisciplinary Bioscience and Bioengineering, Pohang University of Science and Technology, Pohang, Korea; 3 Division of IT Convergence Engineering, Pohang University of Science and Technology, Pohang, Korea; 4 Division of Integrative Biosciences and Biotechnology, Pohang University of Science and Technology, Pohang, Korea; 5 Biotechnology Research Center, Pohang University of Science and Technology, Pohang, Korea; Saint Louis University, United States of America

## Abstract

Successful viral infection requires intimate communication between virus and host cell, a process that absolutely requires various host proteins. However, current efforts to discover novel host proteins as therapeutic targets for viral infection are difficult. Here, we developed an integrative-genomics approach to predict human genes involved in the early steps of hepatitis C virus (HCV) infection. By integrating HCV and human protein associations, co-expression data, and tight junction-tetraspanin web specific networks, we identified host proteins required for the early steps in HCV infection. Moreover, we validated the roles of newly identified proteins in HCV infection by knocking down their expression using small interfering RNAs. Specifically, a novel host factor CD63 was shown to directly interact with HCV E2 protein. We further demonstrated that an antibody against CD63 blocked HCV infection, indicating that CD63 may serve as a new therapeutic target for HCV-related diseases. The candidate gene list provides a source for identification of new therapeutic targets.

## Introduction

Viruses are major human pathogens that cause millions of deaths every year [1]. For instance, hepatitis C virus (HCV), a causative agent of hepatitis, liver cirrhosis, and hepatocellular carcinoma, alone affects more than 170 million people worldwide [2]. Identifying the human genes involved in the early steps of HCV infection will increase our scientific understanding of the HCV life cycle and help to discover valuable therapeutic targets for HCV-related diseases.

A number of genome-wide approaches have been developed in recent decades to uncover host proteins required for the proliferation of viruses. For example, small interfering RNA (siRNA) screens, yeast two-hybrid screens, and microarray analyses have been applied to more fully understand host–virus interactions [3], [4], identify genes functionally related to viral infection, and establish which cellular proteins physically interact with viral proteins. Although the potential of systems biology approaches to identify host proteins required for the viral infections is widely appreciated, previous genome-wide approaches often predict many unprioritized candidate genes, making it difficult to select appropriate host genes for further validation, and additional host proteins related to virus infections remain to be identified. Thus, various computational analysis-based integration approaches should be explored for better understanding of the virus-host interactions [5]–[8].

Many cellular proteins have been reported to participate in the early steps of HCV infection. Among the proteins that have been proposed to act as HCV receptors are CD81 [9], low density lipoprotein receptor (LDLR) [10], scavenger receptor class B type 1 (SCARB1) [11], claudin 1(CLDN1) [12], claudin 6 and 9 (CLDN6 and 9) [13] and, most recently, occludin (OCLN) [14]. The catalytic subunit of cAMP-dependent protein kinase (PRKAC α/β/γ), which modulates the subcellular localization of CLDN1 at the plasma membrane, is essential for viral receptor activity [15]. Binding of HCV to CD81 triggers RAC1/CDC42-mediated actin rearrangement, which is a prerequisite for virus entry [16]. HCV enters the cell via clathrin-mediated endocytosis [17].

In addition to these previously identified proteins, there are likely to be many more host proteins required for the early steps of HCV infection. Therefore, we undertook an integrative-genomics approach to identify host factors required for the early steps in HCV infection. To take full advantage of available resources related to HCV infection, we integrated information from three types of protein attributes: (1) HCV–human protein associations, (2) co-expression with human genes related to the early steps of HCV infection, and (3) association with the protein interaction network of the tight junction-tetraspanin web. Our approach relies on the fact that a host factor is more likely to be involved in HCV infection if its involvement is supported by multiple lines of evidence. In the data integration, we devised a novel method to rank the probability of host protein involvement in HCV infection by weighing each data set. The accuracy of the prediction was further evaluated by testing the effects of knocking down the expression of seven genes predicted to be related to HCV infection. The products of four genes (TJP1, 14-3-3 β, GLUT4, and CD63) out of the seven genes were newly confirmed to participate in the early steps of HCV infection. Among the four genes, we further investigated the role of CD63 in HCV infection and discovered that CD63 directly interacts with HCV E2 protein, which is essential for viral-receptor binding and virus entry. Furthermore, we showed that an antibody against CD63 blocked HCV infection in a dose-dependent manner. It is now revealed for the first time that human protein CD63 as a potential therapeutic target for HCV-related diseases. We provide the list of candidate genes as a resource for identification of host proteins participating in the early steps of HCV infection, which may be useful for drug development required for the treatment of HCV-related diseases.

## Materials and Methods

### Data Sources

Computational methods are fully described in Methods S1. All methods and materials were downloaded in January 2009. We analyzed three types of datasets. To identify genes co-expressed with a query set, we used the GEMMA (www.bioinformatics.ubc.ca/Gemma) database of publicly available microarray studies [18]. We also collected physical interactions of HCV with human proteins by downloading results of genome-wide yeast two-hybrid screens of binding between HCV viral proteins and human proteins [19], curating pathogen–human interaction databases, and manually searching PubMed for literature-based HCV–human protein interactions. Within these HCV–human protein interactions, we only considered interactions mediated by HCV viral protein E1 and/or E2 to obtain HCV entry-specific interactions. We derived the tight junction-tetraspanin web-specific interaction network from comprehensive human protein interaction datasets, which integrated eight existing mammalian protein-protein interaction databases that filtered out low-confidence interactions [20]. For comparison of prediction performance, we used a gene prioritization method applying sequence similarity, literature mining, and domain information to the integrated data [21].

### Data Integration

To integrate evidence from various datasets, we used the Liptak–Stouffer’s weighted Z combination method, which is a type of meta-analysis method for calculating overall Z-score that accounts for both the differential quality of each dataset and correlations among datasets [22]. The null hypothesis is that genes required for the early step of HCV infection will have average score in each feature. The alternative hypothesis is that genes required for the early step of HCV infection have higher scores than the average score in each feature. The weighted Z combination method integrated Z-scores of genes in each features. The total strength of a given gene in functional linkage derived from multiple features was calculated as the weighted sum of individual Z-scores as
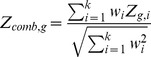
where *k* is the number of features, *Z_g,i_* is the *Z* -score of the gene *g* in the *i* ^th^ feature, and *w_i_* is the weight of the *i* ^th^ feature. The weights were assigned according to the following formula:



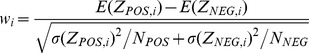
where *Z_POS,i_* is a set of *Z*-scores of gene *g* of the positive set in the *i* ^th^ feature, and *Z_NEG,i_* is a set of *Z*-scores of gene *g* of the negative set in the *i* ^th^ feature. The average value of set Z is represented as *E*(·), and the standard deviation is represented as *σ*(·). *N_POS_* and *N_NEG_* represent the number of genes in positive and negative sets, respectively. A high value of *W_i_* indicates that the *Z*-scores of positive set genes are higher than those of negative set genes. We independently tested the performance of *Z*-scores for each features, and then selected the integration approach that maximized the area under the ROC curve.

### Production of Infectious HCV

RNAs for production of HCVcc were synthesized by in vitro transcription using T7 polymerase (Stratagene 600124). The plasmid pJFH1-5A-Rluc containing an infectious HCV sequence with the Renilla luciferase reporter [23] in the NS5A gene was linearized with the restriction enzyme *Xba*I and then used as a template for transcription. The plasmid Jc1 E2 FLAG containing an infectious HCV sequence with the FLAG epitope in the E2 gene was linearized with the restriction enzyme *Mlu1* [24] and then used as a template for transcription. Huh7.5.1 cells were transfected with RNA as previously described [25]. At 3–5 d post-transfection virus containing supernatants were collected, filtered (0.45 µm), and used to infect naïve cells for 3 hrs. Cell monolayers were then washed twice with PBS and further cultivated for 48–72 hrs, as indicated, prior to analysis of HCV infection. HCVcc infectious titers (focus forming units [FFU]/ml) were determined as described previously [25].

### Production of HCV Psudoparticles (HCVpp)

HCVpp were generated as previously described [26]. Briefly, 293T cells were cotransfected with a plasmid encoding the envelope-deficient HIV genome pNL4-3.Luc.R_.E_ and a plasmid expressing one of the following viral glycoproteins: E1/E2 of HCV strain Con1 (1b) or G protein of stomatitis virus (VSV-G). Medium was replaced with DMEM/3% FBS after 6 hrs of incubation. Supernatants were collected at 48 hrs after cultivation, clarified by centrifugation, and then used in pseudoparticle infection experiments.

### RNA Purification and qRT-PCR

Total RNA was extracted using the TRI reagent (Invitrogen) from HCV infected Huh 7.5.1 cell. Total RNA (2 µg) was reverse transcribed using Improm II reverse transcriptase (Promega), and the cDNA was subjected to real-time PCR analysis for quantification using Sybr premix Ex *Taq* (Takara). Primer sequences for reverse transcription-PCR and real-time PCR were as follows: HCV, 5′-GTCTAGCCATGGCGTTAGTA-3′ and 5-CTCCCGGGGCACTCGCAAGC-3′; glyceraldehyde-3-phosphate dehydrogenase (GAPDH), 5′-TGCACCACCAACTGCTTAG-3′ and 5′-GAGGCAGGGATGATGTTC-3′.

### Immunoprecipitation

JC1 E2 FLAG virus (a gift from Ralf Bartenschlager) infected cell were lysed using the Triton lysis buffer [40 mM HEPES (pH 7.5), 100 mM KCl, 1 mM EDTA, 1 mM PMSF, 0.1% Triton X-100]. The lysates were clarified by centrifugation at 14,000×*g* for 15 min. Anti-FLAG monoclonal antibody and control monoclonal antibody conjugated with agarose resin was incubated with lysates at 4°C for 2 hrs. Precipitates were washed three times with lysis buffer and analyzed by Western blotting.

### Protein Expression and Purification

To construct a plasmid expressing the extracellular loop 2 of CD63 (CD63 EC2), a part of CD63 gene corresponding to nucleotides 307–609 was amplified from a human liver cDNA library with a primer pair 5′-CGGGATCCGCTGGCTATGTGTTTAGAG-3′ (forward) and 5′-CCGGAATTCCTACACATTTTTCCTCAGCCA-3′ (reverse). The PCR-amplified DNA was treated with *BamH*I/*EcoR*I and cloned into the corresponding sites of pGEX-4T3 to generate pGEX-4T-3-CD63EC2. To construct a plasmid expressing the large extracellular loop of CD81 (CD81 LEL), a part of CD81 gene corresponding to nucleotides 337–603 was amplified from a human liver cDNA library with a primer pair 5′- CGCGGATCCTTTGTCAACAAGGACCAGATCG-3′ (forward) and 5′-CCGGAATCC TCACTTCCCGGAGAAGAGGTCAT-3′ (reverse). The PCR-amplified DNA was treated with *BamH*I/*EcoR*I and cloned into the corresponding sites of pGEX-4T3 to generate pGEX-4T-3-CD81 LEL. CD63 EC2-GST and CD81 LEL-GST fusion proteins were expressed in *E. coli* strain BL21 by adding 1 mM Isopropyl-β-D-thiogalactopyranoside (IPTG) when the cell density reached 0.5 OD_600 nm_. After incubating cells at 37°C for additional 6 hrs, cells were harvested and resuspended in lysis buffer [20 mM Na-phosphate (pH 7.5), 300 mM NaCl, 1 mM PMSF, 1 mM β-mercaptoethanol, 1% Triton X-100). GST-fusion proteins were allowed to bind to glutathione Sepharose 4B resin (Amersham-Pharmacia Biotech) in lysis buffer at 4°C for 2 hrs. Resin-bound proteins were eluted with elution buffer [50 mM Tris-Cl (pH 8.0), 10 mM GSH] and dialyzed against phosphate-buffered saline (PBS). To construct a plasmid expressing E2 luminal domain of HCV genotype 2a, a part of pJFH1 cDNA clone corresponding to 1138–2186 nucleotides downstream of the initiation codon was amplified by PCR with a primer pair (5′-GGCACCACCACCGTTGGAG-3′ and 5′-CCACTCCCATCGAACGACG-3′) and a template plasmid pJFH-1. PCR-amplified DNAs were treated with *Nhe1/Hind3* and cloned into the corresponding sites of p425-GPD containing 6xHis and FLAG tag to construct p425-GPD-E2. E2 proteins were expressed in yeast strain PBN204 by transforming yeasts with plasmid p425-GPD-E2. Yeast cells were harvested at 1.0 OD_600 nm_ and resuspended in lysis buffer [20 mM Na-phosphate (pH 7.5), 300 mM NaCl, 1 mM PMSF, 1% Triton X-100]. E2-His-FLAG proteins were allowed to bind to a Talon Metal affinity resin (Clontech Laboratories, Inc. 635502) at 4°C for 2 hrs. The resin-bound proteins were eluted by 200 mM Imidazole in lysis buffer. The eluted proteins were allowed to bind to an ANTI-FLAG M2 affinity gel (Sigma Aldrich A2220) in lysis buffer at at 4°C for 2 hrs, eluted by 100 ug/ml of 3X FLAG peptide (Sigma Aldrich F4799) in lysis buffer, and dialyzed in phosphate buffered saline (PBS).

### GST Pull-down Assays

GST pull-down assays were performed using recombinant GST, GST-CD81 LEL, GST-CD63 EC2, and 6xHis-FLAG-E2. Following incubation of GST fusion proteins (10 ng) and 6xHis-FLAG-E2 (20 ng) in 1 mL of 0.05% tween-20 in PBS for 30 min at 4°C, Glutathione Sepharose 4B resin was added to the mixture and incubated for 1 hour and 30 min. The resin was washed 4 times with incubation buffer, and resin-bound proteins were resolved by 10% SDS-PAGE. The resin-bound proteins were analyzed by Western blotting.

## Results

### Genome-wide Surveys used in the Integrative Analysis

**Figure 1:**
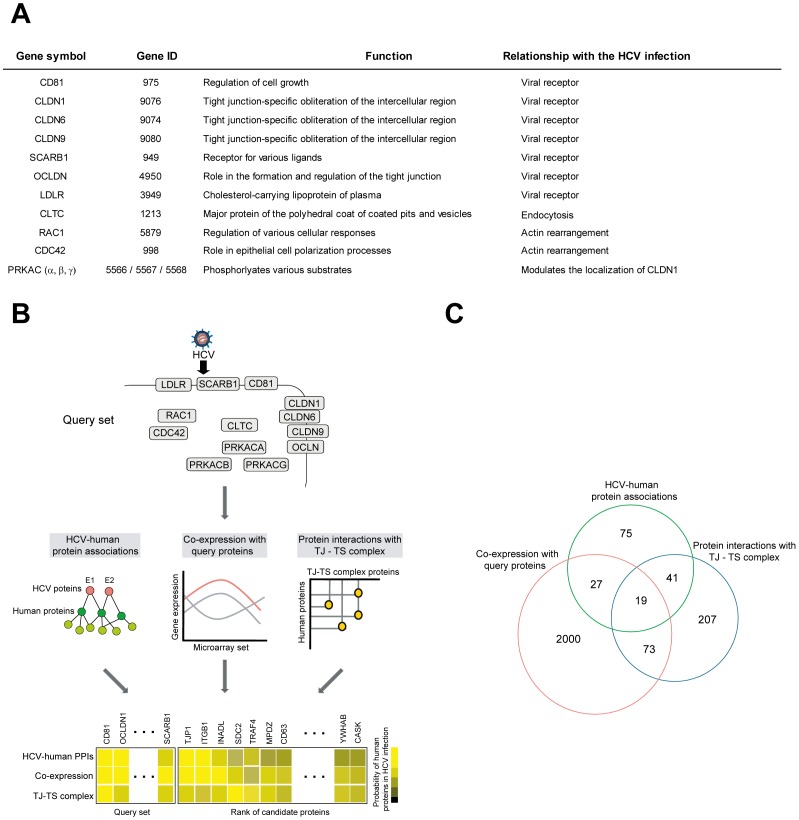
Outline of the procedure for predicting proteins involved in the early steps of HCV infection. (A) List of the thirteen host proteins involved in the early steps of HCV infections. (B) *Upper panel:* In the training step, human genes known to be involved in the early steps of HCV infection were selected as a query set. *Middle panel:* The features strongly related to the early steps of HCV infection were analyzed, and the gene sets known to be involved in each feature were collected. Features used: HCV–human protein association data, co-expression with the query set, and physical interactions with tight junction or tetraspanin web. *Bottom panel:* The weight factor of each data set was assigned according to the correlation of the query set obtained using a meta-analysis. The test proteins were assigned a probability of being related to HCV entry based on the summation of each weight factor in the test step. Finally, proteins were ranked by their feature scores based on the functional similarity with the query set. High-scoring proteins are more tightly connected to the query set and are likely to be new candidates for involvement in early steps of HCV infection. (C) Overlap analysis of gene sets identified by each feature.

In this study, we focused on the interaction between HCV and host factors at the early steps of HCV infection. These steps are associated with various viral and cellular events, including the initial binding of HCV to the host cell surface, internalization of virion particle (endocytosis), vesicle trafficking, fusion of the viral envelope with the host endosomal membrane, and priming for virus replication [27], [28]. Many human genes are known to participate in the early steps of HCV infection, hereafter referred to the “query set” (**Figure 1A**). The query set includes proteins required for viral adsorption and entry (CD81; CLDN-1, -6, and -9; SCARB1; OCLN; LDLR; and CLTC) and proteins that modulate cellular functions during HCV infection (RAC1; CDC42; and PRKAC α/β/γ isoforms.

To predict novel human genes involved in the early steps of HCV infection, we exploited three features that are potentially related to viral infection of the host cell. The first feature was HCV and human protein interactions. We identified the proteins that directly interact with or form complexes with viral proteins including HCV E1 and/or E2 (viral envelope proteins), which are the major contributors to HCV–host cell interactions during the fusion of virus and cell membrane. These proteins, whose interactions have been confirmed by yeast two-hybrid screening and literature-curated protein interactions [19], [29], [30], were designated “primary interaction partners”. Next, we created a list of proteins that interact with primary interaction partners. These proteins, designated “secondary interaction partners”, are potentially important because proteins identified as “indirectly interacting” based on high-throughput screens often turn out to be direct but transient interactors [31], [32]. The interacting proteins were assembled from the human protein interactome constructed from eight pre-existing databases [20]. A total of 162 human proteins were included in the group designated “E1/E2-associating proteins”, which included both primary and secondary interaction partners (**File S1**). Among 155 secondary interaction partners, 12 proteins were predicted to interact with HCV viral proteins such as NS3 and NS5A (**Table S1**). We speculate that these proteins may participate in priming for viral RNA replication. The second feature exploited was co-expression with human genes involved in HCV infection. It has been shown that functionally related genes tend to be co-expressed [33]. Using the GEMMA program to analyze co-expression profiles [18], we identified 2,119 genes that are co-expressed at least three times with one of the query genes; these were designated “co-expressed genes” (**File S2**). To build the co-expression profile, we analyzed hundreds of datasets from publically available microarray databases. The third feature we used was the protein-interaction network of the tight junction and tetraspanin web (tight junction – tetraspanin web network). Many proteins that participate in the early steps of HCV infection are known to be involved in tight junction or tetraspanin web. For example, CD81, a member of the tetraspanin web, and the tight junction proteins CLDN1 and OCLN are among the known HCV receptors [12], [14], [16]. Therefore, we expected that the protein interaction with participants of the tight junction – tetraspanin web network as one of the key features in predicting proteins required for the early steps in HCV infection. Based on the comprehensive human protein interactome database [20], we constructed a tight junction – tetraspanin web protein interaction network containing 340 human proteins; these were designated “tight junction – tetraspanin web network” (**Figure S1** and **File S3**). The three features together called 2,442 genes as candidate genes for host proteins participating in the early steps of HCV infection (**Figure 1C** and **File S4**). The pairwise overlaps were modest, and the percentages of shared genes ranged from 2% to 13%. We expected that each feature reflects different biological characters and plays the complementary role.

### Prediction of Proteins Involved in the Early Steps of HCV Infection

Human proteins involved in the early steps of HCV infection were predicted with a data integrative approach according to the following steps. First, individual proteins obtained different scores depending on the number of associations with E1/E2 proteins, the number of genes co-expressed with the query proteins, and the number of interactions with a tight junction – tetraspanin web protein. Second, weight factors assigned to each of the three factors were dependent on the discriminative power between query set (positive set) and negative set (**Figure S2**). Leave-one-out cross-validation was used to measure the prediction performance (see details in **MATERIALS AND METHODS**). At every step, one of the proteins in the query set was set aside from the training set; 100 randomly selected genes were used as a negative control set. This process was repeated 1,000 times. Using this approach, we calculated true-positive and false-positive rates. In order to improve the prediction performance, we integrated the three features into a single model based on a meta-analysis [34].

We found that the predictive performance of our method was higher than that of any one of the individual features alone. As shown in the rank receiver operating characteristic (ROC) curves, the integrated feature improved performance, as measured by the true-positive rate versus false-positive rate (**Figure S4A**). We also plotted the area under the ROC curve (AUC) as a standard indicator of prediction performance, and obtained an AUC value of 0.86, which is higher than that of all individual features (**Figure S4B**). An AUC value of 1.0 indicates perfect performance, whereas a value of 0.5 indicates random prediction. Notably, our approach yielded a higher AUC value than the gene-prioritization method [21], which was designed to prioritize disease-associated genes and pathway-related genes through integration of multiple data sources (**Figure S4**). Although the gene prioritization method integrates more features, our approach performed better. This higher prediction performance was due, at least in part, to the suitability of the features chosen, which are highly relevant to the prediction of human proteins related to HCV infection (e.g., tight junction – tetraspanin web network).

### Functional Analyses of the Predicted Proteins

**Figure 2:**
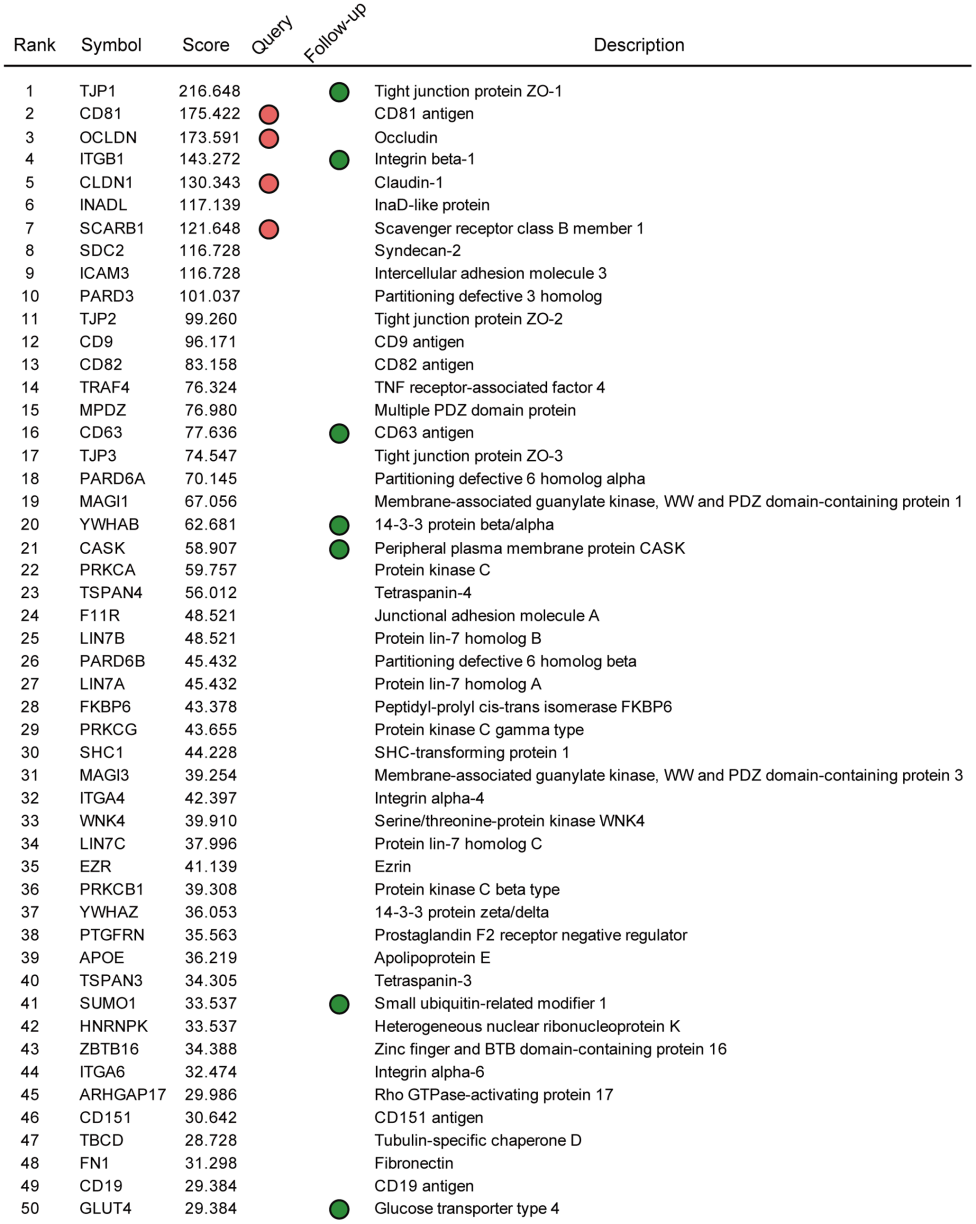
List of the top 50 ranked proteins predicted to be involved in the early steps of HCV infection. Red circles, proteins in the query set; green circles, proteins selected for further experimental validation.

To understand biological functions of human proteins targeted by HCV, we analyzed the enrichment of biological processes and signaling pathways of the top 100 ranked proteins predicted to be involved in HCV infection. We found that the majority of them are novel proteins which are not previously been implicated in mediating HCV infection, although the list includes four query proteins (red circles) (**File S4** and **Figure 2**).

**Figure 3:**
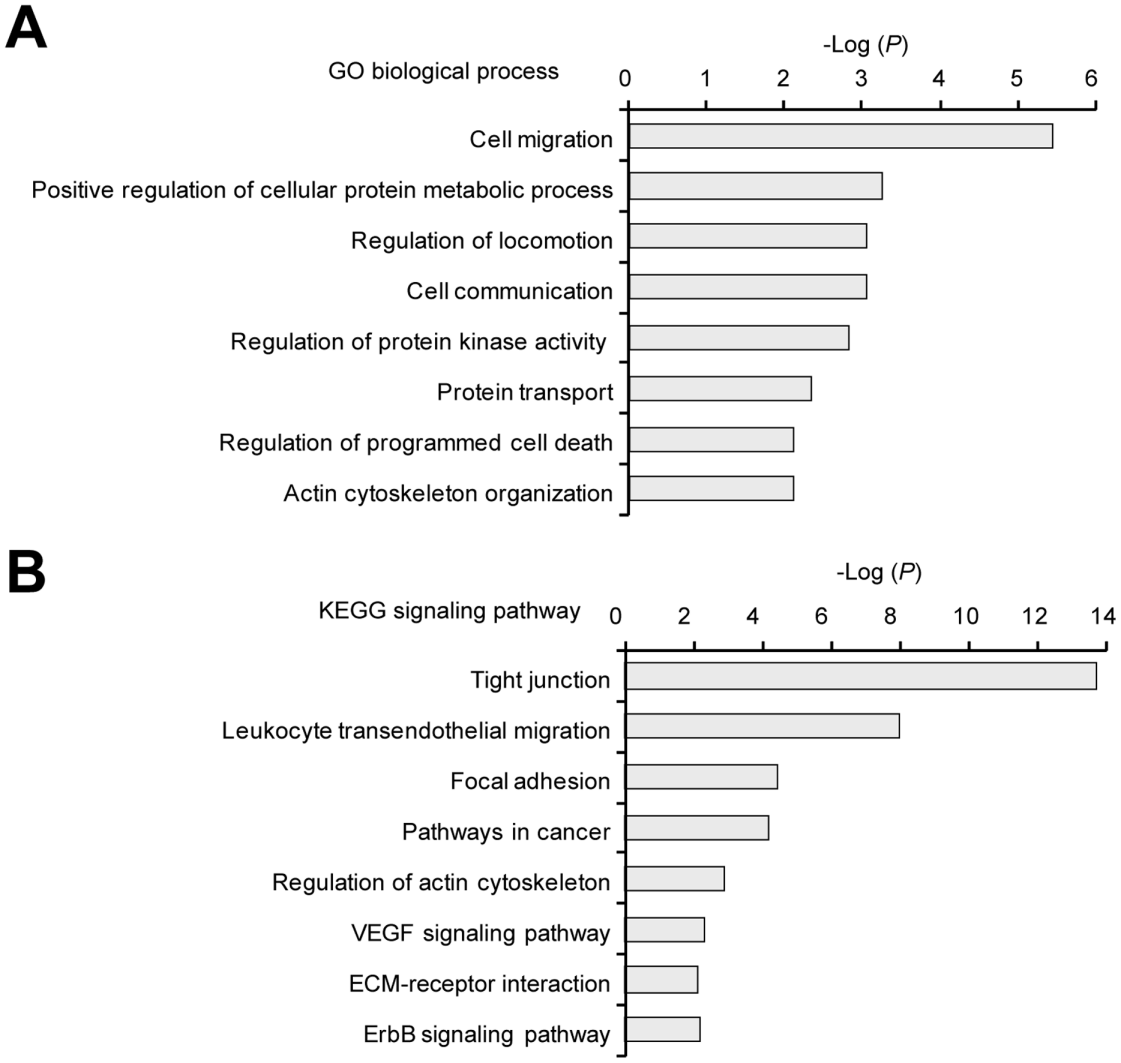
Functional characterization of the top 100 ranked proteins predicted to be involved in the early steps of HCV infection. (A) Statistically significant overrepresentation of functional terms based on mapping of Gene Ontology (GO) biological process to the top 100 ranked human proteins (*P*<1.0×10^−2^ after correlation for Benjamini). (B) Significantly overrepresented KEGG signaling pathways (*P*<1.0×10^−2^ after correlation for Benjamini).

We analyzed the functional enrichment of top 100 ranked proteins from Gene Ontology (GO) biological processes and KEGG signaling pathway (**File S5** and **File S6**; see detail in **Method S1**). We discovered that enriched biological functions of highly ranked proteins were similar to those of proteins known to be involved in the early steps of other virus infections [35], such as regulation of kinase activity and protein transport (*P*<5.0×10^−3^ after correlation for Benjamini multiple test [36]; **Figure 3A**). Significantly enriched signaling pathways included tight junctions, regulation of cell migration, apoptosis, and actin cytoskeleton (*P*<1.0×10^−3^) after correlation for Benjamini multiple test [36]; **Figure 3A**). We also observed that highly ranked proteins participated in the same biological functions suggested by recent genome-wide screens of human proteins required for HCV infection [37]–[39]. For example, enriched biological functions involved in HCV inflection were focal adhesion, and ErbB signaling (**Figure 3B**). The top 100 ranked proteins identified here warrant further study as novel proteins that act early during HCV infection, with likely involvement in associated functions of virus entry and HCV infection. We provide the list of complete candidate genes containing 2,442 genes (**File S4**) as a resource for identification of host proteins participating in the early steps of HCV infection, which may be useful for drug development in the treatment of HCV-related diseases.

### Experimental Confirmation of the Predicted Genes

We selected seven proteins from the top 100 ranked proteins for experimental confirmation of the prediction using the following criteria: (i) High scoring proteins were preferentially selected. (ii) Proteins previously reported to be involved in HCV infection were excluded. For example, APOE, identified here, has been shown to affect the HCV infectivity [40]. (iii) Proteins localized to the plasma membrane or cytoplasm were selected because the early steps of HCV infection occur in these subcellular loci. (iv) When a family of functionally related proteins was predicted, a representative protein was experimentally tested. For example, TJP1 was selected, although TJP2 and TJP3 were also predicted to be involved in HCV infection. (v) Structural proteins (cytoskeletal and related proteins) and proteins of unknown functions were excluded. Using these criteria, we selected seven proteins–TJP1, integrin β1, CD63, 14-3-3 β, CASK, SUMO1, and GLUT4 (green circles in **Figure 2**)–from among the top 100 ranked proteins. All of the seven selected proteins were associated with only HCV E1 and/or E2 not with other HCV proteins. According to the network topology analysis, TJP1, ITGB1 and CD63 were important constituents of the HCV-host protein association network and of the tight junction – tetraspanin web protein network (**Table S2**).

**Figure 4:**
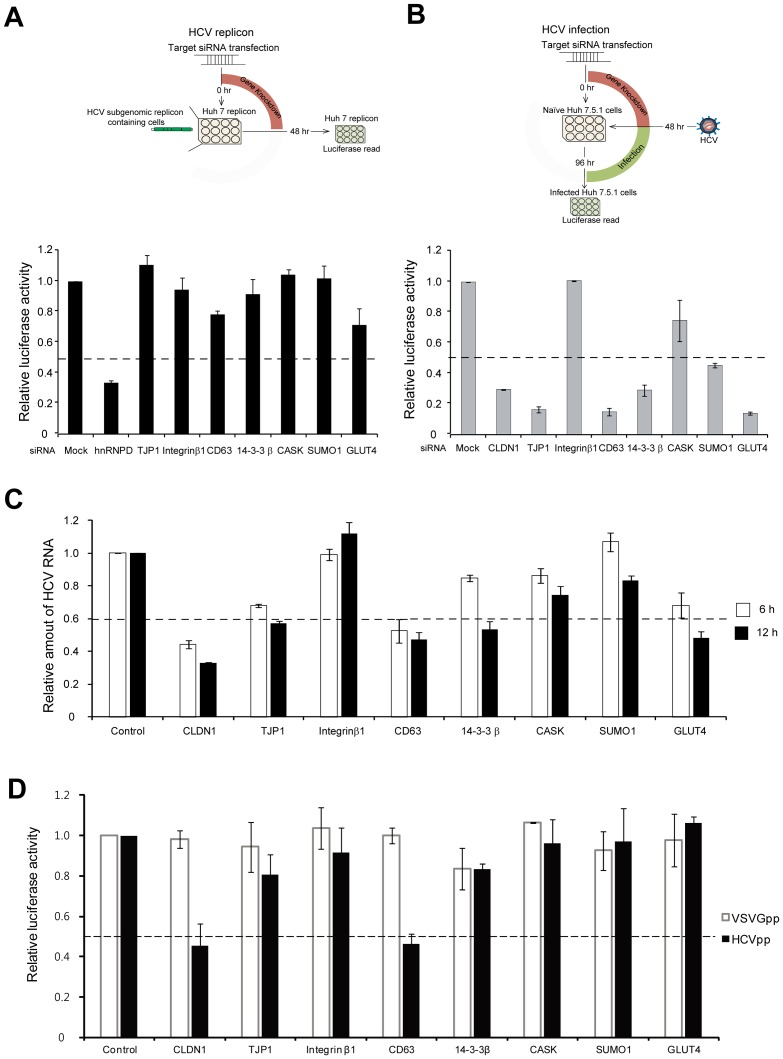
Empirical validation of prediction performance of the integrated approach. (A) Knockdown effects of candidate proteins in HCV replicon. RLuc-replicon cells were transfected with a negative control siRNA or siRNAs against the seven candidate genes. At 48 hrs after transfection, cells were harvested and lysed, and Renilla luciferase activity in the cell lysates was measured. Luciferase activities were normalized to the amounts of proteins in the cell lysates (mean ± s.d. from three independent experiments performed in duplicate). An siRNA against hnRNPD was used as a positive control. The relative luciferase activities in the lysates are depicted by setting the luciferase activity in the control lysate to 1. (B) Knockdown effects of candidate proteins on HCV infection. Huh7.5.1 cells were transfected with a negative control siRNA or siRNAs against the seven candidate genes. An siRNA against CLDN1 was used as a positive control. At 48 hrs after transfection of siRNAs, the cells were infected with JFH1 5A-Rluc virus (MOI of 0.3) and cultivated for an additional 48 hrs. Virus infectivity was monitored by measuring Renilla luciferase activity in cell extracts. The luciferase activities were normalized to the amounts of proteins in the cell extracts (mean ± s.d. from three independent experiments performed in duplicate). The relative luciferase activities in the lysates are depicted by setting the luciferase activity in the control lysate to 1. (C) The relative amounts of HCV RNA in the cells at 6 or 12 hrs after infection. Huh7.5.1 cells were transfected with a negative control siRNA, a positive control siRNA (CLDN1), or siRNAs against the seven candidate genes. At 48 hrs after the siRNA transfections, the cells were infected with JFH1 5A-Rluc virus (MOI of 5). At 6 or 12 hrs after HCV infection, cells were harvested, total RNAs were purified, and the amounts of HCV and GAPDH RNAs were measured by qRT-PCR. The amounts of HCV RNAs were normalized by those of GAPDH RNAs (mean ± s.d. from three independent experiments performed in duplicate). (D) Knockdown effects of candidate proteins on HCV entry. Huh7.5.1 cells were transfected with a negative control siRNA, a positive control siRNA (CLDN1), or siRNAs against the seven candidate genes. At 48 hrs after the siRNA transfections, the cells were infected with HCVpp or VSVGpp, and then cultivated for additional 48 hrs. Infectivities of HCVpp and VSVGpp were monitored by measuring Renilla luciferase activities in the cell extracts, and normalized to the amounts of proteins in the cell extracts. The relative luciferase activities in the lysates are depicted by setting the luciferase activity in the control lysate to 1 (mean ± s.d. from three independent experiments performed in duplicate).

In order to confirm that the predicted proteins participate in the early steps of HCV infection, we investigated the effects of knocking down their expression by treatment with siRNAs. In the first set of experiments, we tested the effects of siRNAs in cells carrying an HCV 1b genotype (con1) subgenomic replicon (NS3-NS5B) containing a Renilla luciferase reporter gene [41]. All of the siRNAs specifically reduced the expression of the target proteins, as judged by Western blotting (**Figure S5**). siRNAs against candidate proteins had no cytotoxic effects (**Figure S6**). None of the siRNAs affected replicon maintenance in the cells (criterion: ≥50%), indicating that these proteins do not play a key role in translation or replication of viral mRNA, which are essential for replicon maintenance (**Figure 4A**). Importantly, siRNA-mediated knockdown of TJP1, CD63, 14-3-3 β, SUMO1 or GLUT4 reduced the infectivity of HCV by more than 50%, whereas knocking down either of the other two proteins (integrin β1 and CASK) did not affect infection of HCV JFH1 5A-Rluc [42] (**Figure 4B**). An siRNA against CLDN1, which is essential for HCV infection [12], was used as a positive control for the HCV infection system, whereas hnRNP D, which is required for HCV replication and translation [43], was used as a positive control for the replicon system. In order to further define the step(s) of HCV proliferation requiring the newly identified proteins, we investigated the effects of knocking down the genes on the levels of viral RNA in the cells at the early stages of infection (6 and 12 hrs after infection). After knocking down the target genes, cells were infected with HCV [5 multiplicity of infection (moi)] for 6 or 12 hrs. The levels of HCV RNA were measured by quantitative RT-PCR (qRT-PCR) after isolation of RNAs in the infected cell at 6 or 12 hrs post infection. HCV entry and uncoating occur until 6 hrs post infection, whereas virus entry-uncoating and priming for replication occur until 12 hrs post infection [27], [28]. Knockdown of CD63, as well as that of the positive control CLDN1, reduced HCV RNA level more than 50% at 6 hrs post infection (**Figure 4C**). On the other hand, knockdown of CLDN1, TJP1, CD63, 14-3-3 β, and GLUT4 reduced HCV RNA level more than 50% at 12 hrs post infection (**Figure 4C**). These data indicate that CD63 is required at the very early stage of HCV infection such as virus entry, but TJP1, 14-3-3 β, and GLUT4 are needed at the later stage(s) of infection such as priming for viral RNA replication. We further confirmed the roles of the newly identified genes in virus proliferation using HCV pseudoparticles that are suitable for testing the entry step of HCV infection [26]. Consistently with the qRT-PCR assays, knockdown of CLDN1 and CD63 reduced the infectivity of HCV pseudoparticles more than 50% (**Figure 4D**). On the other hand, knockdown of other genes did not affect the infectivity of HCV pseudoparticles (**Figure 4D**). The infectivity of VSVG pseudoparticles was not affected by knockdown of any genes tested (**Figure 4D**). Taken the data together, we concluded that CD63 protein is needed for the HCV entry step, and that TJP1, 14-3-3 β, and GLUT4 are needed for RNA uncoating and/or priming of RNA replication. These data confirm that our data-integrative approach efficiently identifies novel proteins associated with HCV infection. The role of CD63 in HCV infection was analyzed further (below).

### CD63 Facilitates HCV Entry through a Direct Interaction with HCV E2

We found for the first time that CD63 participates at the entry step of HCV infection. According to our data-integrated approach, CD63 interacts with 15 proteins in the tight junction-tetraspanin web protein network, and is a secondary interaction partner of E1/E2. CD63 is a tetraspanin protein containing two extracellular loops and three cytoplasmic domains. CD63 participates in clathrin-mediated endocytosis [44]. Although the precise function of CD63 remains unknown, it has been characterized as a marker for activation or differentiation of various cell types.

**Figure 5:**
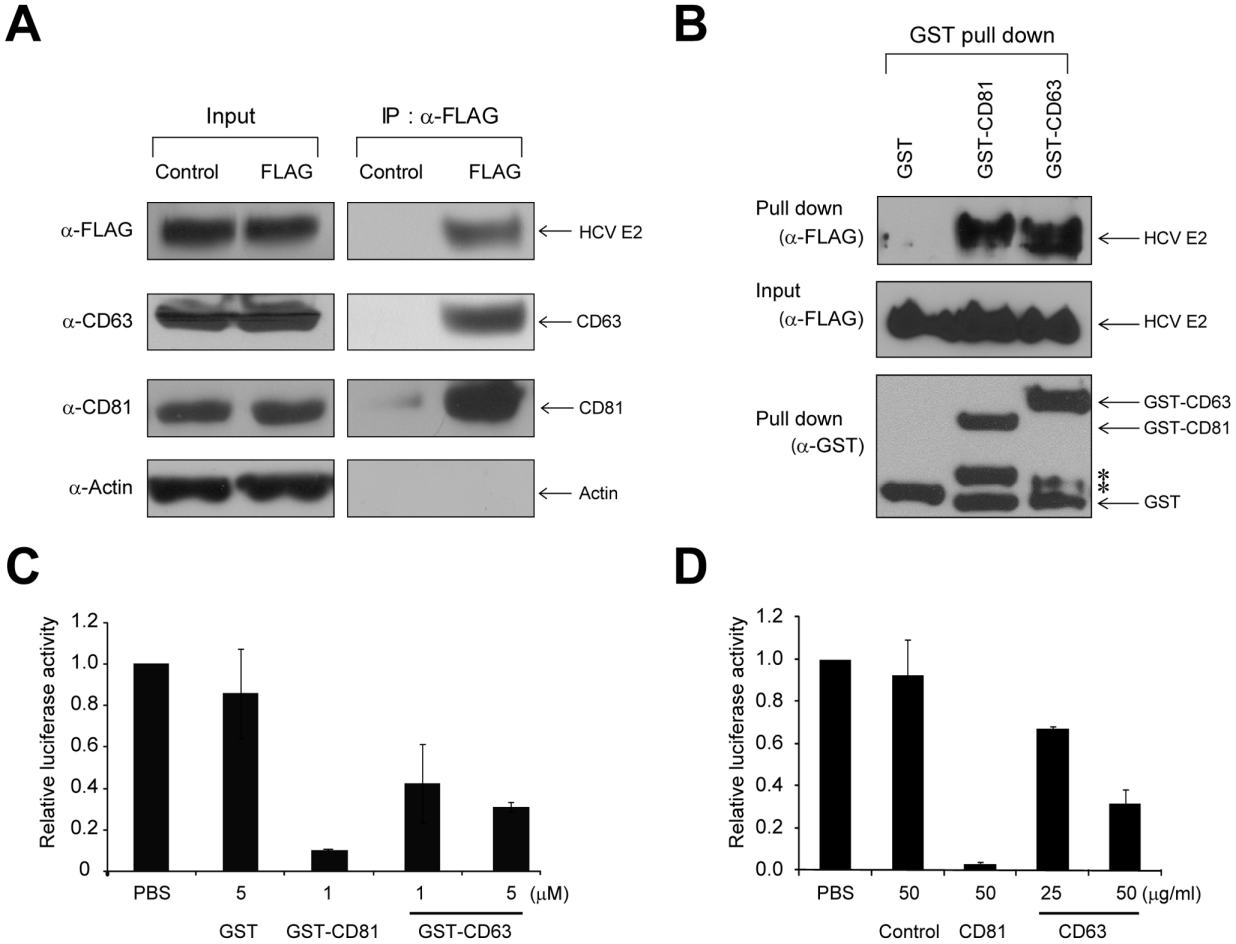
CD63 participates in HCV entry through a direct interaction with HCV E2. (A) Co-immunoprecipitations of CD63 and CD81 by HCV E2. Extracts of Huh 7.5.1 cells, which were infected with JC1 E2 FLAG virus (MOI 0.3) for 72 hrs, were analyzed by Western blotting to detect the indicated proteins before and after immunoprecipitations with a FLAG antibody or a control mouse antibody. Actin is a negative control. (B) GST pull-down assays with purified proteins. GST-fused CD63 EC2 (GST-CD63) and GST-fused CD81 LEL (GST-CD81) proteins were expressed in *E. coli* and then purified (Methods). FLAG-tagged HCV E2 (FLAG-E2) proteins were expressed in yeast and then purified (Methods). After incubating the purified FLAG-E2 proteins with GST, GST-CD81 or GST-CD63 proteins for 2 hrs at 4°C, GST, GST-fusion proteins, and their associated proteins were precipitated with GSH Sepharose 4B. The resin-bound proteins were analyzed by Western blotting with antibodies against GST or FLAG. Degraded forms of GST-CD63 and GST-CD81 (indicated by asterisks) were also precipitated by the GSH resin. (C) Effect of a polypeptide corresponding to the CD63 EC2 domain on HCV infection. JFH1 5A-Rluc virus was incubated with GST or GST-CD63 for 2 hrs at 4°C. Huh7.5.1 cells were then inoculated with the virus–polypeptide mixtures by incubating for 3 hrs at 37°C, and the cells were further cultivated for 48 hrs. Virus infectivity was monitored by measuring Renilla luciferase activities in cell extracts, and normalized to the amounts of proteins in cell extracts (mean ± s.d. from three independent experiments performed in duplicate). The relative luciferase activities in experimental lysates to that in the control lysate (PBS) are depicted. (D) Effect of an anti-CD63 antibody (BEM-1 from Santa Cruz Biotechnologies) on HCV infection. Huh7.5.1 cells were pre-incubated with a negative control mouse IgG1, a positive control anti-CD81 antibody, or an anti-CD63 antibody at the indicated concentrations for 1 hour at 37°C, and then inoculated with JFH1 5A-Rluc virus (MOI of 0.3). The cells were cultivated for additional 48 hrs, and then Renilla luciferase activities in cell lysates were measured and normalized to the amounts of proteins in lysates (mean ± s.d. from three independent experiments performed in duplicate). The relative luciferase activities in experimental lysates to that in the control lysate (PBS) are depicted.

To investigate the interaction between HCV E2 and CD63, we performed immunoprecipitation experiments using JC1 E2 FLAG virus containing a FLAG-tag in the E2 protein [24]. Huh-7 cells infected with JC1 E2 FLAG virus were lysed in Triton X-100 lysis buffer which prevents CD81-CD63 interaction by disrupting tetraspanin microdomains [45]. Endogenous CD63 was co-precipitated with a FLAG antibody recognizing E2-FLAG protein (**Figure 5A**). A positive control CD81, which directly interacts with HCV E2, was co-immonoprecipitated by the FLAG antibody. We further tested whether CD63 directly interacts with HCV E2. GST pull-down experiments with purified proteins revealed that FLAG-tagged E2 was co-precipitated with GST-CD63 but not with a negative control GST protein (**Figure 5B**, compare GST and GST-CD63 lanes in the α-FLAG panel). GST-CD81 LEL was used as a positive control of the direct interaction with E2. These results indicate that CD63 directly interacts with HCV E2. The role of CD63 in HCV infection was further investigated by monitoring the effect of purified GST-CD63 on HCV infection. Preincubation of GST-CD63 with HCV blocked infection of HCV in a dose-dependent manner (**Figure 5C**). No inhibition of HCV infection was observed with a negative control GST protein, and stronger inhibition was observed with a positive control GST-CD81. Moreover, pre-incubation of host cells with an antibody against CD63 also blocked HCV infection in a dose-dependent manner (**Figure 5D**). No inhibition of HCV infection was observed with a negative control antibody, and stronger inhibition was observed with a positive control antibody against CD81 (**Figure 5D**). Taken together, these data suggest that CD63 participates in HCV infection through a direct interaction with HCV E2, and CD63 may be a novel target for developing anti-HCV drugs.

## Discussion

To identify novel human proteins that facilitate the early steps of HCV infection, we devised a novel approach that exploited information on HCV–human protein interactions, co-expression with human genes related to HCV infection, and association with the protein interaction network of the tight junction-tetraspanin web. In this approach, three features were evaluated and candidate genes were prioritized by multiple lines of features. We demonstrated that incorporating these three features significantly enhanced the prediction performance compared with any single feature, and we observed robust prediction performance irrespective of the coverage of protein interaction network (**Figure S7**). Moreover, we could assign ranks to the predicted proteins based on the number of protein–protein interactions within a feature and on the number of query genes co-expressed with the gene. This approach gave high ranks to some of proteins known to participate in HCV infection. However, this approach gave low ranks to some genes known to participate in the early steps of HCV infection such as CLTC and LDLR (ranking 827 and 1659, respectively) due to the lack of interaction with tight junction or tetraspanin web proteins. This indicates that our integrative approach may not cover all of the genes involved in HCV infection. Nevertheless, we consider our method is useful to find the novel human genes involved in HCV infection. We also anticipate that a similar approach could be applied to identify proteins associated with the key pathways of other viral infections.

We evaluated the reliability of the prediction by empirically analyzing the effects of knocking down the predicted proteins on HCV infection. Four proteins–TJP1, 14-3-3 β, GLUT4 and CD63 were shown to be involved in the early steps of HCV infection (**Figure 4**). Among these proteins, we further analyzed the role of CD63 in HCV infection since CD63 has a high potential to serve as a target for developing anti-HCV drugs. We demonstrated that the E2 protein directly interacts with CD63. HCV infection was inhibited by a purified polypeptide corresponding to the extracellular domain 2 (EC2) of CD63 and by an anti-CD63 antibody (**Figure 5**). Moreover, knockdown of CD63 prevented infection of HCV pseudoparticles (**Figure 4**). These results suggest that CD63 participates in the HCV entry step. CD63, a member of the tetraspanin protein family, is a ubiquitously expressed protein that is localized to the cell surface and the endosomal system. The lysosome-targeting motif of CD63 is required for its association with AP-2, which is involved in clathrin-mediated endocytosis from the plasma membrane [44], and with AP-3, which is responsible for redistribution of CD63 from recycling endosomes to lysosomes [46]. Therefore, CD63 may participate in endocytosis and lysosome targeting of HCV-containing vesicles, a step that follows interaction of virus with viral receptors, such as SCARB1 and CD81. The detailed role of CD63 in HCV infection remains to be elucidated.

In this study, we hypothesized that novel human proteins that are involved in the early steps of HCV infection are tightly connected at the molecular level through protein interactions or co-expression with proteins known to be involved in an early step of HCV infection. We have demonstrated that it is possible to construct a model that accurately predicts novel host factors. Using this method, it is possible to reduce the amount of time and effort required for identifying genes responsible for HCV infection compared to other approaches, such as high-throughput screening. Here, we focused on the early steps of HCV infection. However, this approach could easily be extended to other steps in HCV life-cycle or other viruses by integrating relevant biological features. Similar approaches could be applied to discover genes that participate in other biological processes, including pathogen infection. We thus expect that many more genes will be characterized using such data-integrative methods, and anticipate that this approach will lead to a more thorough understanding of virus–host relationships.

## Supporting Information

Figure S1 Protein interaction network of the tight junction and tetraspanin web proteins. Proteins are represented by nodes and physical interactions by edges. Red node: annotated tight junction protein, blue node: annotated tetraspanin web protein, and yellow node: interacted proteins with tight junction or tetraspanin web proteins.
(DOCX)

Figure S2 Weight factors of individual feature.
(DOCX)

Figure S3 Characteristics of the HCV – human protein associations (**A**) and protein interactions with tight junction-tetraspanin web protein network (TJ-TS complex) (**B**). The analyses were performed by the Cytoscape plugin, NetworkAnalyzer.
(DOCX)

Figure S4 Performance of the data-integrative approach for predicting proteins involved in early steps of HCV infection. Individual datasets and the integrated model (integration) were evaluated for their performance in predicting genes previously known to participate in the early steps of HCV infection using a cross-validation approach. (**A**) Rank ROC curves obtained from the validation of the early steps of HCV infection. (**B**) The AUC values are obtained for all individual features and the integration method after fusing all individual features are shown. The AUC value is a standard measurement of predictability that ranges from 0.5 for random prediction to 1 for perfect prediction.
(DOCX)

Figure S5 Western blot analyses of candidate proteins before and after siRNA treatments. Western blotting of actin protein was performed as a negative control.
(DOCX)

Figure S6 Cytotoxic effects of siRNA treatment. Huh 7.5.1 cells were transfected with various siRNAs for 48 hours. Cytotoxicity was measured with a ToxiLight BioAssay Kit (mean ± s.d. from three independent experiments performed in duplicate). No signs of toxicity were observed from the cells treated with the siRNAs.
(DOCX)

Figure S7 Performance of the data-integrative approach for predicting proteins involved in early steps of HCV infection. Integrated model (integration) and with comprehensive protein interaction network (Kim *et al*., 2011) were evaluated for their performance in predicting genes previously known to participate in the early steps of HCV infection using a cross-validation approach. (**A**) Rank ROC curves obtained from the validation of the early steps of HCV infection. (**B**) The AUC values obtained from all individual features or from the integration method after fusing all individual features. The AUC value is a standard measurement of predictability that ranges from 0.5 for random prediction to 1 for perfect prediction.
(DOCX)

Table S1 List of secondary interaction partners associated with other HCV proteins.
(DOCX)

Table S2 Network topology of six selected proteins. *SUMO1 was not included in the data of either HCV-Human protein association or TJ-TS complex.
(DOCX)

Methods S1 (DOCX)

File S1 Listing of human proteins associated with HCV viral protein E1 and/or E2.
(XLSX)

File S2 Listing of human genes coexpressed with query genes.
(XLSX)

File S3 Listing of tight junction and tetraspanin web proteins.
(XLSX)

File S4 List of the human proteins predicted to be involved in the early steps of HCV infection.
(XLSX)

File S5 Gene Ontology (biological process) enrichment analysis of top 100 proteins.
(XLSX)

File S6 KEGG enrichment analysis of top 100 proteins.
(XLSX)
