# Supplementary figures and images for: Discovery of Cellular Proteins Required for the Early Steps of HCV Infection Using Integrative Genomics
Source: PLoS One. 2013 Apr 12;8(4):e60333. doi: 10.1371/journal.pone.0060333 (PMC3625227; doi:10.1371/journal.pone.0060333)

**Figure S1**.


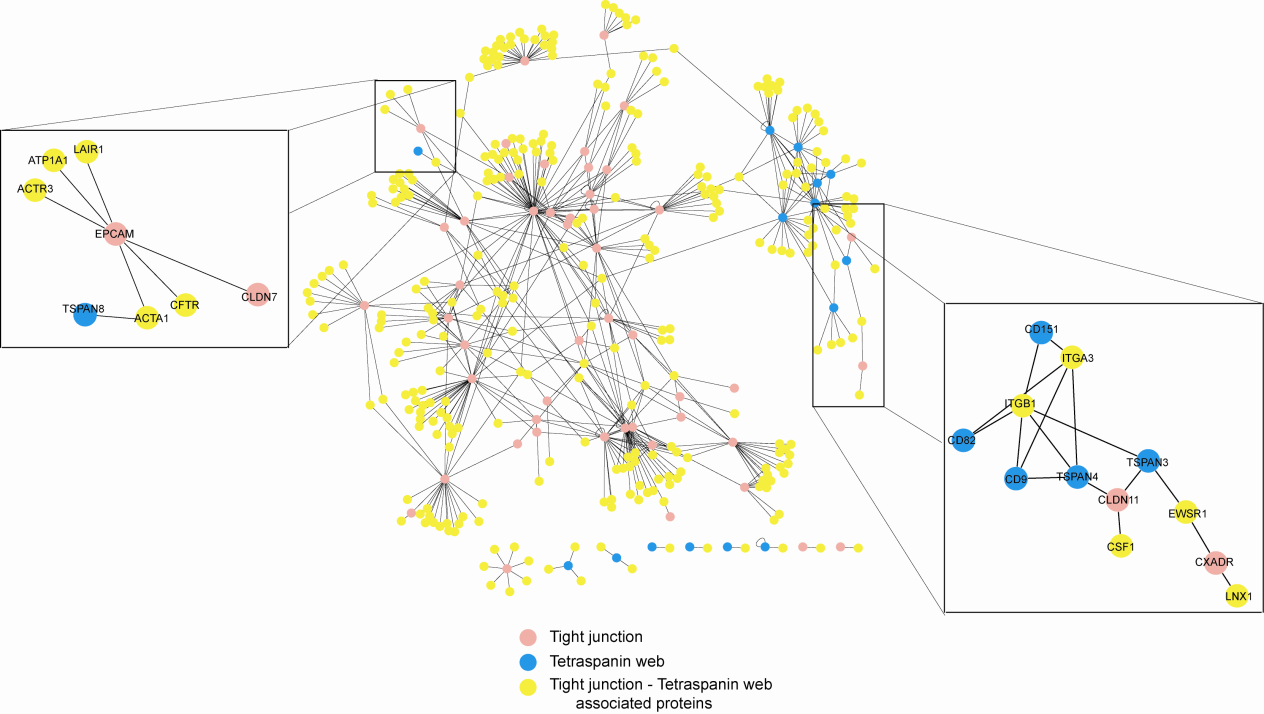

Supplement: Figure S1 — Protein interaction network of the tight junction and tetraspanin web proteins. Proteins are represented by nodes and physical interactions by edges. Red node: annotated tight junction protein, blue node: annotated tetraspanin web protein, and yellow node: interacted proteins with tight junction or tetraspanin web proteins. (DOCX) [file pone.0060333.s001.docx]

**Figure S2**.


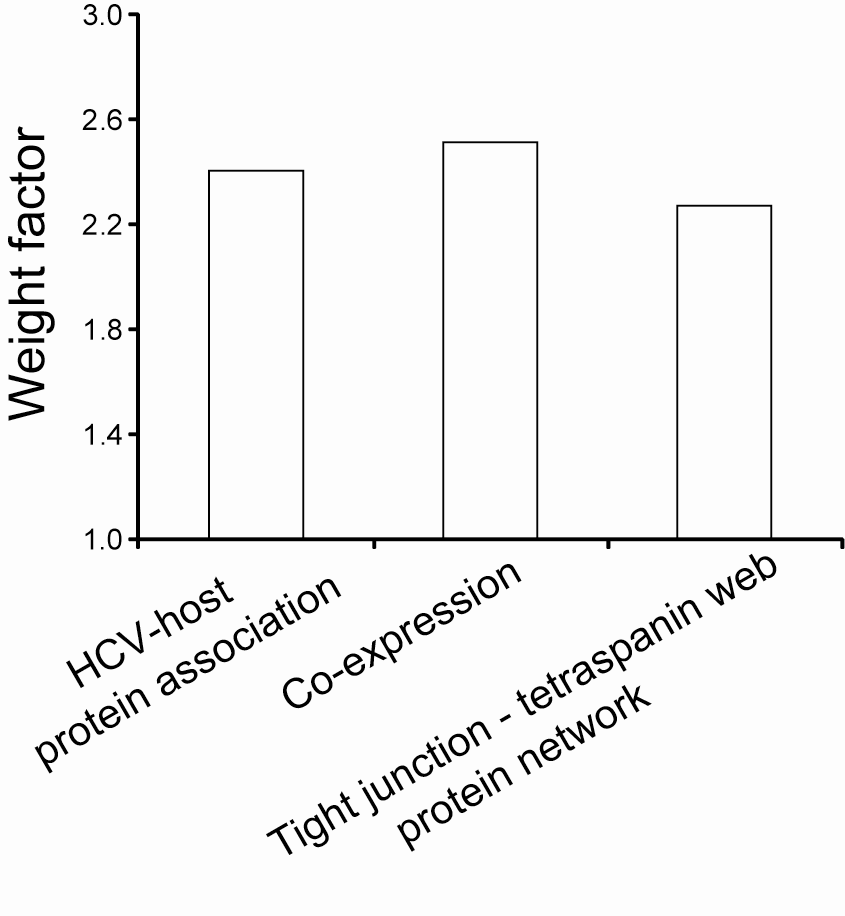

Supplement: Figure S2 — Weight factors of individual feature. (DOCX) [file pone.0060333.s002.docx]

**Figure S3**.


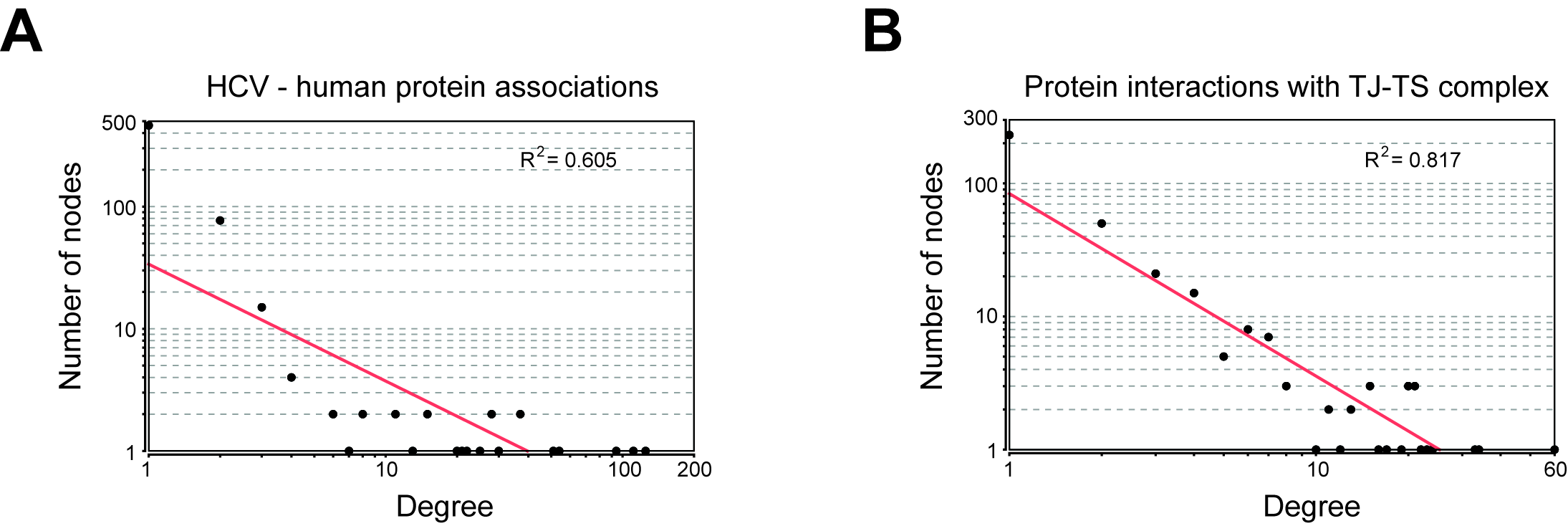

Supplement: Figure S3 — Characteristics of the HCV – human protein associations (A) and protein interactions with tight junction-tetraspanin web protein network (TJ-TS complex) (B). The analyses were performed by the Cytoscape plugin, NetworkAnalyzer. (DOCX) [file pone.0060333.s003.docx]

**Figure S4**.


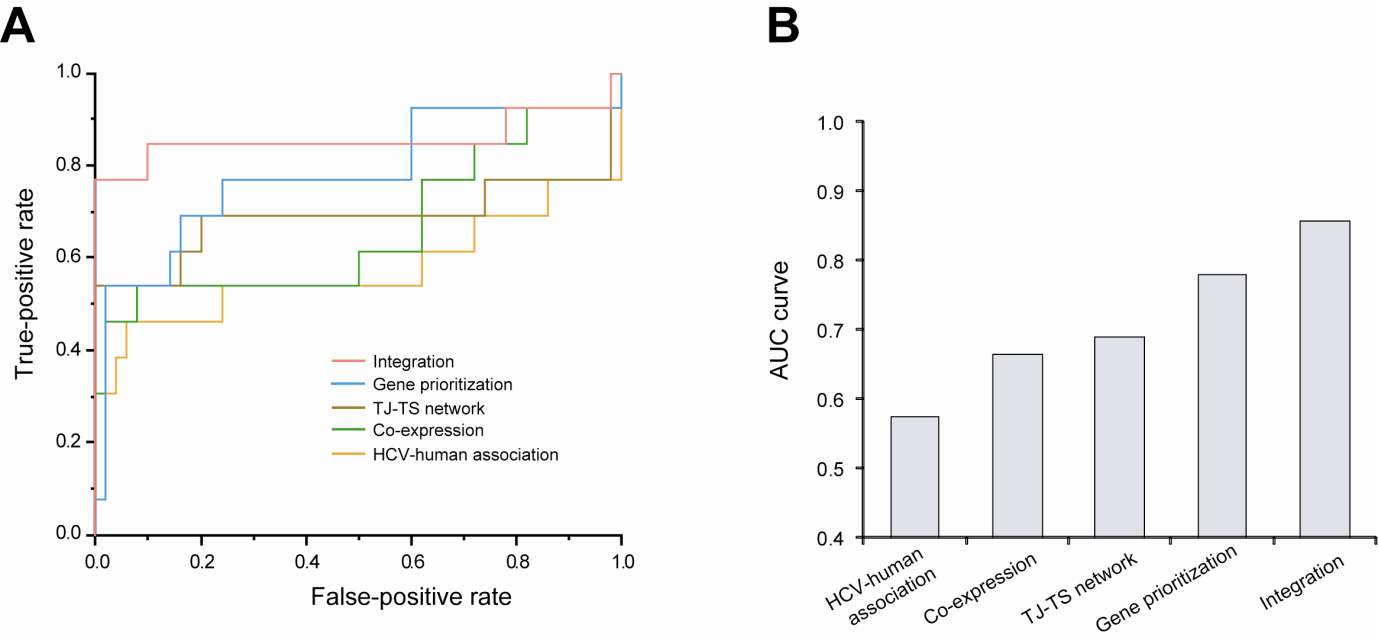

Supplement: Figure S4 — Performance of the data-integrative approach for predicting proteins involved in early steps of HCV infection. Individual datasets and the integrated model (integration) were evaluated for their performance in predicting genes previously known to participate in the early steps of HCV infection using a cross-validation approach. (A) Rank ROC curves obtained from the validation of the early steps of HCV infection. (B) The AUC values are obtained for all individual features and the integration method after fusing all individual features are shown. The AUC value is a standard measurement of predictability that ranges from 0.5 for random prediction to 1 for perfect prediction. (DOCX) [file pone.0060333.s004.docx]

**Figure S5**.


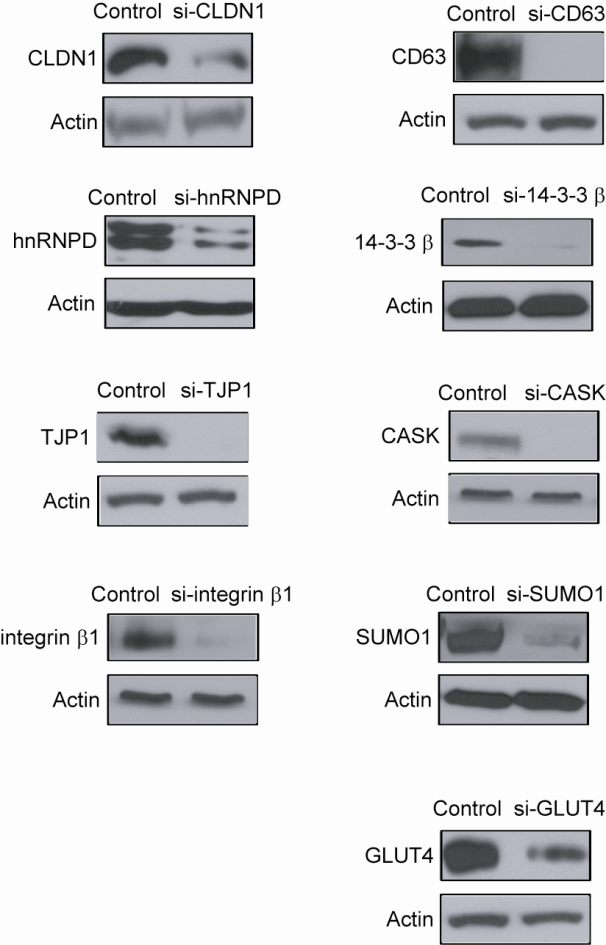

Supplement: Figure S5 — Western blot analyses of candidate proteins before and after siRNA treatments. Western blotting of actin protein was performed as a negative control. (DOCX) [file pone.0060333.s005.docx]

**Figure S6**.


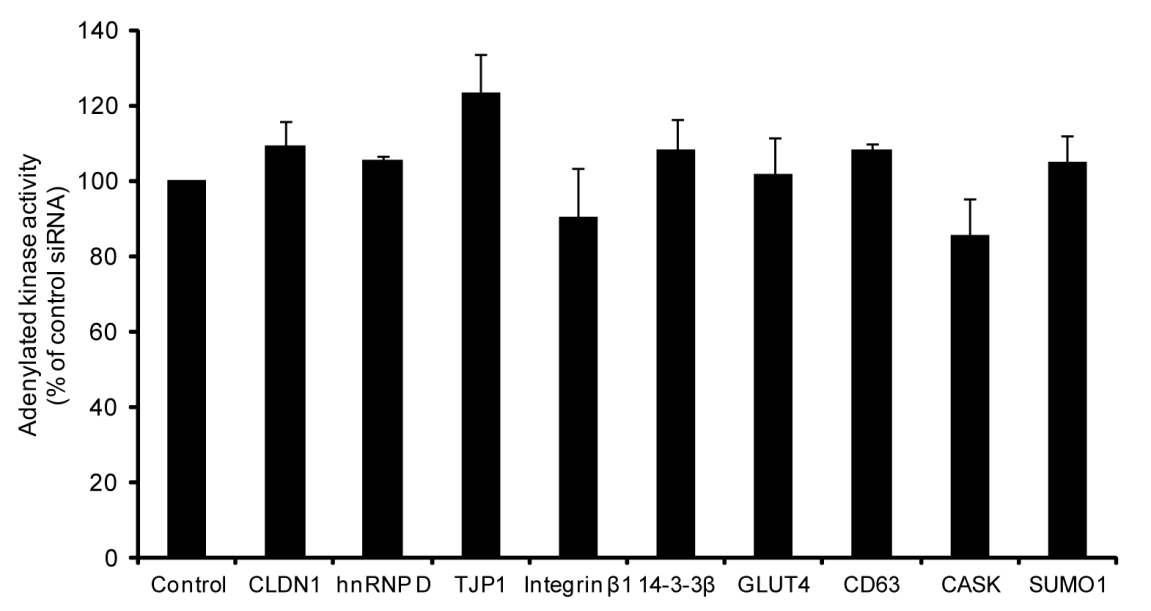

Supplement: Figure S6 — Cytotoxic effects of siRNA treatment. Huh 7.5.1 cells were transfected with various siRNAs for 48 hours. Cytotoxicity was measured with a ToxiLight BioAssay Kit (mean ± s.d. from three independent experiments performed in duplicate). No signs of toxicity were observed from the cells treated with the siRNAs. (DOCX) [file pone.0060333.s006.docx]

**Figure S7**

**
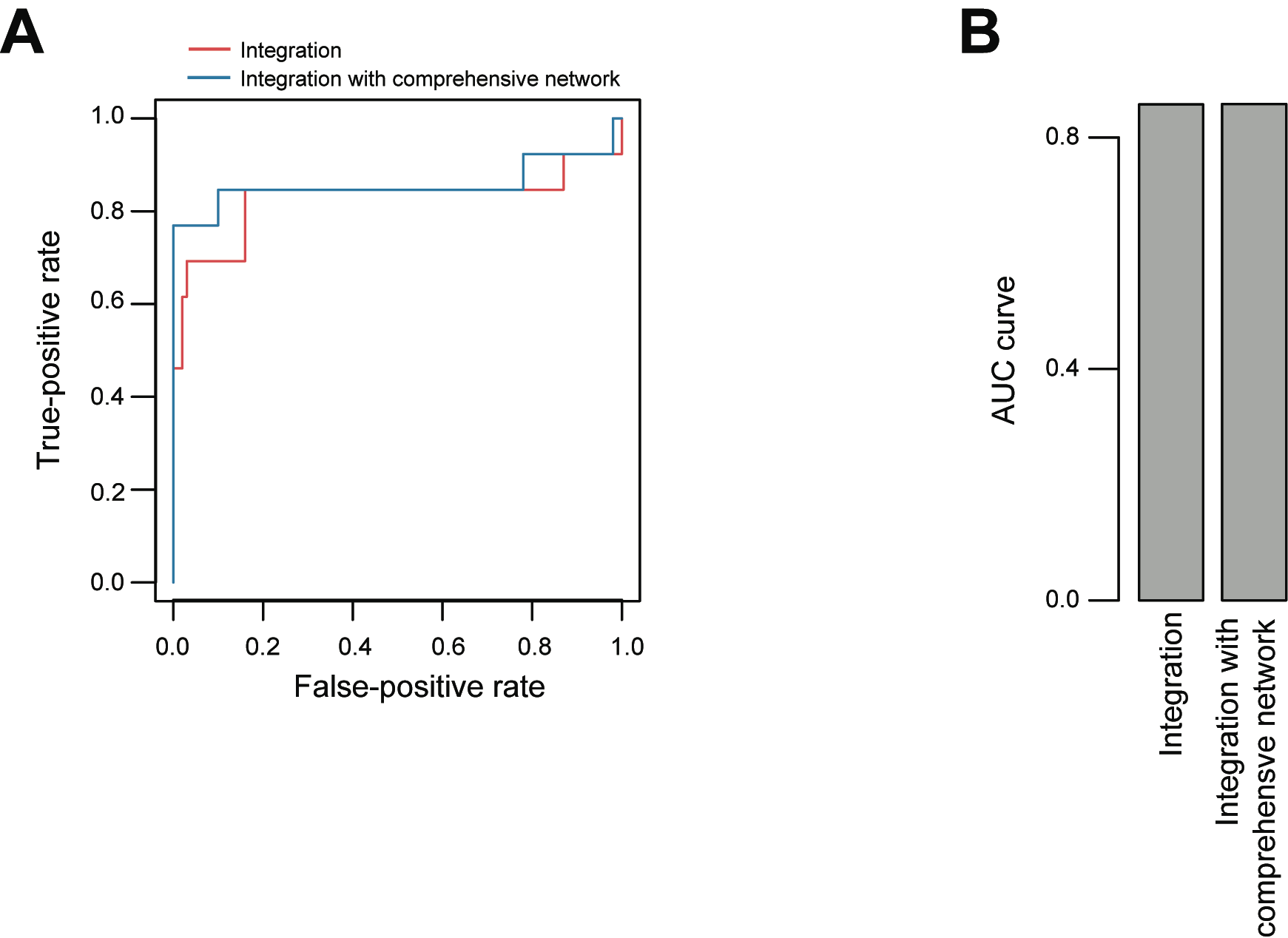
**

Supplement: Figure S7 — Performance of the data-integrative approach for predicting proteins involved in early steps of HCV infection. Integrated model (integration) and with comprehensive protein interaction network (Kim et al., 2011) were evaluated for their performance in predicting genes previously known to participate in the early steps of HCV infection using a cross-validation approach. (A) Rank ROC curves obtained from the validation of the early steps of HCV infection. (B) The AUC values obtained from all individual features or from the integration method after fusing all individual features. The AUC value is a standard measurement of predictability that ranges from 0.5 for random prediction to 1 for perfect prediction. (DOCX) [file pone.0060333.s007.docx]
