# Supplementary material for: Discovery of Cellular Proteins Required for the Early Steps of HCV Infection Using Integrative Genomics
Source: PLoS One. 2013 Apr 12;8(4):e60333. doi: 10.1371/journal.pone.0060333 (PMC3625227; doi:10.1371/journal.pone.0060333)
Supplement: Table S1 — List of secondary interaction partners associated with other HCV proteins. (DOCX) [file pone.0060333.s008.docx]

**Table S1**.

| Viral protein | Entrez ID | Gene symbol |
| --- | --- | --- |
|  |  |  |
| NS5A | 335 | APOA1 |
| F | 462 | SERPINC1 |
| NS3 | 1460 | CSNK2B |
| NS3 | 2192 | FBLN1 |
| NS3 | 2199 | FBLN2 |
| NS3 | 2335 | FN1 |
| NS3 | 1462 | VCAN |
| NS5A | 2274 | FHL2 |
| NS5A | 3683 | ITGAL |
| NS5A | 7046 | TGFBR1 |
| NS5A | 7057 | THBS1 |
| p7 | 2199 | FBLN2 |
