# Supplementary material for: Discovery of Cellular Proteins Required for the Early Steps of HCV Infection Using Integrative Genomics
Source: PLoS One. 2013 Apr 12;8(4):e60333. doi: 10.1371/journal.pone.0060333 (PMC3625227; doi:10.1371/journal.pone.0060333)
Supplement: Table S2 — Network topology of six selected proteins. *SUMO1 was not included in the data of either HCV-Human protein association or TJ-TS complex. (DOCX) [file pone.0060333.s009.docx]

**Table S2**.

| **A**. HCV-human protein associations | | |
| --- | --- | --- |
| Gene symbol | Degree | Betweenness Centrality |
|  |  |  |
|  |  |  |
| TJP1 | 2 | 0.000 |
| ITGB1 | 94 | 0.261 |
| CD63 | 2 | 0.000 |
| YWHAB  (14-3-3β) | 1 | 0.000 |
| CASK | 1 | 0.000 |
| GLUT4 | 1 | 0.000 |
|  |  |  |
|  |  |  |
| **B**. Protein interactions with TJ-TS complex | | |
| Gene symbol | Degree | Betweenness Centrality |
|  |  |  |
|  |  |  |
| TJP1 | 60 | 0.396 |
| ITGB1 | 7 | 0.030 |
| CD63 | 15 | 0.045 |
| YWHAB  (14-3-3β) | 9 | 0.093 |
| CASK | 3 | 0.003 |
| GLUT4 | 3 | 0.014 |
