# Supplementary material for: Discovery of Cellular Proteins Required for the Early Steps of HCV Infection Using Integrative Genomics
Source: PLoS One. 2013 Apr 12;8(4):e60333. doi: 10.1371/journal.pone.0060333 (PMC3625227; doi:10.1371/journal.pone.0060333)
Supplement: Methods S1 — (DOCX) [file pone.0060333.s010.docx]

**Supplementary Materials and Methods**

**HCV–Human protein association data**

We downloaded all datasets used in this study in January 2009. We compiled 162 experimentally detected and manually curated protein–protein interactions between HCV and human proteins from the primary literature [[1](#_ENREF_1),[2](#_ENREF_2)] and four interaction databases: the Molecular INTeraction database [[3](#_ENREF_3)], the Biomolecular Interaction Network Database [[4](#_ENREF_4)], IntAct [[5](#_ENREF_5)], and Reactome [[6](#_ENREF_6)]. We also manually searched the interactions between HCV and human proteins in PubMed (covering publications until January 2009). We considered the subcellular localizations of human proteins to obtain specific interaction partners for the early steps of HCV infection and ultimately selected 7 human proteins that interacted with E1 and/or E2. To define secondary interaction partners, we used a comprehensive protein–protein interaction network that integrates eight existing interaction databases [[7](#_ENREF_7)]. For measuring the quality of HCV-human protein network, network topology was analyzed by Cytoscape Plugin, NetworkAnalyzer [[8](#_ENREF_8)] (**Figure S3A**).

**The tight junction-tetraspanin web specific network**

We compiled a list of human proteins containing annotations for “tight junction” or “tetraspanin”, as listed in **File S3**. These proteins were used as seed proteins for constructing the tight junction-tetraspanin web specific network. Because low coverage and high false-positive rates are limitations in protein–protein interaction datasets, we used a comprehensive protein–protein interaction network that integrates eight existing interaction databases [[7](#_ENREF_7)]. We collected only high-quality interaction datasets by removing low confidence interactions through various filtering steps: exclusion of data sets predicted by high-throughput methods, orthologous interactions from lower organisms than human, and *in silico* methods. For measuring the quality of HCV-human protein network, network topology was analyzed by Cytoscape Plugin, NetworkAnalyzer [[8](#_ENREF_8)] (**Figure S3B**). For the comprehensive analyses, we applied the recent integrated protein interaction network from Kim *et al*, [[9](#_ENREF_9)] comprising 101,777 interactions between 11,043 human proteins.

**Proteins co-expressed with the query set**

We identified proteins co-expressed with the query set using the GEMMA database [[10](#_ENREF_10)], a publicly available database for the meta-analysis of microarray studies. The authors collected previously published microarray sets and, to reduce false-positives, presented co-expressed genes reported in at least three datasets. We used the query set as an input and collected proteins that were co-expressed with at least one query protein.

**Prioritization of genes underlying the early steps of HCV infection**

We identified proteins involved in the early steps of HCV infection using the Endeavour program [[11](#_ENREF_11)], which integrates data obtained through various means, such as literature mining, functional annotation, EST expression, pathway information, transcription motif, and sequence similarity. This analysis provided the 200 candidate genes based on their similarities to the input set.

**Performance evaluation**

We tested the performance of our method using leave-one-out cross-validation. Several measures were used to evaluate performance. All were derived from the numbers of true positives (TP: human proteins known to be involved in early steps of HCV infection predicted as proteins participating in early steps HCV infection), false positives (FP: proteins in the random set predicted as proteins participating in early steps HCV infection), true negatives (TN: proteins in the random set predicted not to be proteins participating in early steps HCV infection), and false negatives (FN: proteins known to be involved in early steps of HCV infection predicted not to be proteins participating in early steps HCV infection). To avoid any training set bias that could potentially arise, we randomly chose 100 test proteins from the human genome. This procedure was repeated 1,000 times.

**Gene Ontology/pathway-enrichment analyses**

We obtained GO and KEGG categories from the Database for Annotation, Visualization, and Integrated Discovery (DAVID; <http://david.abcc.ncifcrf.gov/>) [[12](#_ENREF_12),[13](#_ENREF_13)] to compute enrichments of both GO biological processes and KEGG signaling pathways. To optimize annotation specificity and comprehensiveness, we selected GO terms that were assigned to fewer than 40 but more than five human genes (terms assigned to more than 40 genes are too general, and terms assigned to less than five genes are too specific). In the GO enrichment analysis, 14,116 genes were considered in the background. Significant GO terms were manually curated for visual representation of the GO data; a complete list of significantly enriched GO terms divided into functional groups is provided in **File** **S5**. *P*-values of functional enrichment analyses obtained using an accumulated hypergeometric distribution function followed by application of a Benjamini multiple test correction was calculated for the primary hit lists. The same analysis was also applied to hit lists from the KEGG signaling pathway (**File** S**6)**. 5,085 genes were considered in the background in the enrichment analysis of KEGG signaling pathway.

**Antibodies and chemicals**

The indicated antibodies against the following proteins were utilized for Western blotting, immunoprecipitation and neutralization: anti-GST [[14](#_ENREF_14)]. anti-CASK (MAB5230) from Chemicon; anti-CD63 (sc-5275, sc-65975), anti-GLUT4 (sc-53566), anti-integrin β1 (sc-8978), SUMO1 (sc-5308), anti-TJP1 (sc-10804), anti-14-3-3β (sc-628) and control mouse IgG (sc-2025) from Santa Cruz Biotechnologies. anti-CD81 (555675) from BD Pharmingen. anti-hnRNP D (07-260) from Upstate Biotechnology. anti-FLAG(F3165) from SIGMA-ALDRICH.

**Cell culture**

Huh7.5.1 cells were cultivated in Dulbecco's modified Eagle's medium (DMEM; Gibco) supplemented with 10% fetal bovine serum (Clontech). Huh7.5.1 cells containing a subgenomic replicon (NS3-NS5B) of HCV genotype 1b (con1) with a Renilla luciferase gene and a neomycin phosphotransferase gene [[15](#_ENREF_15)] were cultivated in DMEM supplemented with 10% fetal bovine serum and antibiotic G418 (500 µg/ml; Calbiochem).

**RNA interference**

The following siRNAs (Bioneer Inc., Korea) were used in this study:

CASK siRNA: 5’-GGUAUUGGAAGAAAUUUCA dTdT-3’

CD63 siRNA: 5’-GUUCUUGCUCUACGUCCUC dTdT-3’

GLUT4 siRNA: 5’-CAGAUAGGCUCCGAAGAUG dTdT-3’

Integrin β1 siRNA: 5’-GCGCAUAUCUGGAAAUUUG dTdT-3’

SUMO1 siRNA: 5’-CUGGGAAUGGAGGAAGAAG dTdT-3’

TJP1 siRNA: 5’-AAAUGAGGAUUAUCUCGUCUU dTdT-3’

14-3-3 β siRNA: 5’-CCCUGAAUGAAGAGUCUUA dTdT-3’

hnRNP D siRNA: 5’-GAUUGACGCCAGUAAGAACdTdT-3’

siRNAs were transfected into Huh7.5.1 cells and Rluc-replicon containing cells using Oligofectamine (Invitrogen) according to the manufacturer’s instructions.

**Western blot analysis**

Proteins were resolved by sodium dodecyl sulfate-polyacrylamide gel electrophoresis (SDS-PAGE), transferred to a nitrocellulose membrane, and probed with the indicated antibodies. The fold-dilutions of primary antibodies ranged from 1:200 to 1:1000. Anti-mouse, anti-goat, or anti-rabbit immunoglobulin G was used as a secondary antibody. Protein bands were visualized by enhanced chemiluminescence according to the manufacturer’s instructions (Amersham).

**Cytotoxicity assays**

An adenylate kinase non-destructive cytotoxicity assay was performed utilizing the ToxiLight bioassay kit (Lonza) according to the manufacturer’s standard protocol for adherent cells in 12 well plates.

**Luciferase assay**

Luciferase assays were performed using a dual luciferase assay kit (Promega) according to the manufacturer's instructions.

**Reference**

1. de Chassey B, Navratil V, Tafforeau L, Hiet MS, Aublin-Gex A, et al. (2008) Hepatitis C virus infection protein network. Mol Syst Biol 4: 230.

2. Randall G, Panis M, Cooper JD, Tellinghuisen TL, Sukhodolets KE, et al. (2007) Cellular cofactors affecting hepatitis C virus infection and replication. Proc Natl Acad Sci U S A 104: 12884-12889.

3. Chatr-aryamontri A, Ceol A, Peluso D, Nardozza A, Panni S, et al. (2009) VirusMINT: a viral protein interaction database. Nucleic Acids Res 37: D669-673.

4. Alfarano C, Andrade CE, Anthony K, Bahroos N, Bajec M, et al. (2005) The Biomolecular Interaction Network Database and related tools 2005 update. Nucleic Acids Res 33: D418-424.

5. Kerrien S, Alam-Faruque Y, Aranda B, Bancarz I, Bridge A, et al. (2007) IntAct--open source resource for molecular interaction data. Nucleic Acids Res 35: D561-565.

6. Matthews L, Gopinath G, Gillespie M, Caudy M, Croft D, et al. (2009) Reactome knowledgebase of human biological pathways and processes. Nucleic Acids Res 37: D619-622.

7. Bromberg KD, Ma'ayan A, Neves SR, Iyengar R (2008) Design logic of a cannabinoid receptor signaling network that triggers neurite outgrowth. Science 320: 903-909.

8. Assenov Y, Ramirez F, Schelhorn SE, Lengauer T, Albrecht M (2008) Computing topological parameters of biological networks. Bioinformatics 24: 282-284.

9. Kim J, Kim I, Yang JS, Shin YE, Hwang J, et al. (2012) Rewiring of PDZ domain-ligand interaction network contributed to eukaryotic evolution. PLoS Genet 8: e1002510.

10. Lee HK, Hsu AK, Sajdak J, Qin J, Pavlidis P (2004) Coexpression analysis of human genes across many microarray data sets. Genome Res 14: 1085-1094.

11. Aerts S, Lambrechts D, Maity S, Van Loo P, Coessens B, et al. (2006) Gene prioritization through genomic data fusion. Nat Biotechnol 24: 537-544.

12. Huang da W, Sherman BT, Lempicki RA (2009) Systematic and integrative analysis of large gene lists using DAVID bioinformatics resources. Nat Protoc 4: 44-57.

13. Dennis G, Jr., Sherman BT, Hosack DA, Yang J, Gao W, et al. (2003) DAVID: Database for Annotation, Visualization, and Integrated Discovery. Genome Biol 4: P3.

14. Kim JE, Ryu I, Kim WJ, Song OK, Ryu J, et al. (2008) Proline-rich transcript in brain protein induces stress granule formation. Mol Cell Biol 28: 803-813.

15. Lohmann V, Korner F, Koch J, Herian U, Theilmann L, et al. (1999) Replication of subgenomic hepatitis C virus RNAs in a hepatoma cell line. Science 285: 110-113.
